# Improved filtration method to isolate pure populations of primary bovine endometrial epithelial and stromal cells for immunological studies

**DOI:** 10.1007/s11259-020-09770-3

**Published:** 2020-02-21

**Authors:** Paul Kelly, Anne Barry-Reidy, Amy Brewer, Kieran G. Meade, Cliona O’Farrelly

**Affiliations:** 1 Comparative Immunology Group, School of Biochemistry and Immunology, Trinity Biomedical Sciences Institute, Trinity College Dublin, Dublin, Ireland; 2 Animal & Bioscience Research Department, Animal & Grassland Research and Innovation Centre, Teagasc, Grange, Ireland; 3 School of Medicine, Trinity College Dublin, Dublin, Ireland

**Keywords:** Primary culture, Epithelial cell, Stromal fibroblast, Innate immune response

## Abstract

**Objectives:**

Isolation and culture of distinct primary endometrial cells are key to reliable in-vitro models to investigate the uterine immune response and optimse new disease interventions. Details on the isolation method and purity of distinct cell populations is lacking in currently available protocols leading to inconsistent results across laboratories.

**Methods:**

Bovine endometrial tissue from non-pregnant bovine uteri were collected immediately post-mortem and separated using differential size filtering. Isolations (*n* = 15) yielded an average of 3.1 × 10^5^ ± 0.7 × 10^5^ epithelial cells and 1.88 × 10^6^ ± 5.44 × 10^5^ stromal fibroblasts per uterine horn. Following expansion in culture, the purity of cell populations was confirmed using morphology and positive staining for cytokeratin and vimentin which identifies epithelial and stromal fibroblast populations, respectively. Using PCR, cDNA from both cell populations was negative for CD45, a marker of immune cells.

**Results:**

On challenge with a bacterial PAMP (LPS), epithelial and stromal fibroblasts showed a marked increase in the expression of the inflammatory mediators *IL8*, *IL6*, *S100A8* and *S100A9,* with both cell populations displaying distinct expression profiles. Here we provide a detailed methodology on the culture of primary bovine endometrial epithelial and stromal cells and demonstrate these cells provide a physiologically relevant model for studies of endometrial inflammation and its regulation.

**Electronic supplementary material:**

The online version of this article (10.1007/s11259-020-09770-3) contains supplementary material, which is available to authorized users.

## Introduction

The endometrium, the mucosal lining of the uterus, plays a complex role maintaining a homeostatic environment to allow for successful implantation and pregnancy while tolerating the local microbiome and also protecting against invading pathogens. The bovine endometrium is composed of a single layer of columnar epithelial cells which provide a barrier overlying the stromal matrix (Frandson et al. 2009). While the epithelial cells are in constant interaction with immunogenic material from the external environment and commensal microbiome, during parturition and the subsequent process of tissue remodelling the epithelial layer can be disrupted. This results in stromal fibroblast cells becoming exposed to microbial associated molecular patterns (MAMPs) and endogenous danger associated molecular patterns (DAMPs), thereby activating inflammatory pathways which can result in uterine disease if not resolved. Identifying the inflammatory pathways operating in and between endometrial cells is crucial to understanding the pathology behind uterine disease and the development of targeted therapeutics for local pathology.

In addition to their barrier function, epithelial cells are potent producers of a wide selection of host defence peptides (HDPs) including defensins, cathelicidins and whey acid proteins, as well as pro-inflammatory cytokines and chemokines such as IL-6 and IL-8 (Cronin et al. 2012; Davies et al. 2008; Narciandi et al. 2011; Whelehan et al. 2014). Stromal fibroblast cells also produce pro-inflammatory cytokines and play a role in defence against viral infection (Donofrio et al. 2007). Possession of such a wide repertoire of protective mechanisms demonstrates the pivotal roles epithelial and stromal fibroblast cells play in the early stages of the innate immune response before immune cell recruitment (MacKintosh et al. 2013). However detailed investigation into the innate immune properties of endometrial cells is hindered by difficulty in isolating and culturing these distinct cell populations.

While in-vivo models have classically been used to capture the overall complexity of immune responses, the visualisation of cell type-specific signalling pathways, particularly under controlled stimulation environments, poses a challenge. In addition, in-vivo modelling of bovine immune responses is expensive and restricted by the large inter-animal variation due to extraneous influences (nutrition, housing etc.). In order to examine the immune mechanisms operating within the endometrium, a model that allows us to easily manipulate and control experimental variables while providing quantitative readouts would be useful. Endometrial cell cultures, including the use of cell lines, explants and primary cell populations, have been developed (Borges et al. 2012; Fortier et al. 1988; Herath et al. 2006; Staggs et al. 1998). While cell lines provide a good basis for investigation, they come with limitations including the lack of inter-animal variation provided by primary cultures. In addition, the genetic manipulation require to immortalise a cell results in altered phenotype, function and responsiveness (Kaur and Dufour 2012). The culture of tissue explants provides a potential link between cellular studies and in-vivo models but is restricted by the limited viability of explants in-vitro. Primary cell cultures provide more realistic insight into immune mechanisms, retaining the physiology and genetic makeup of their cellular counterparts in-vivo and demonstrating a level of inter-animal variation which is more reflective of the whole population.

Primary bovine endometrial cell isolation and culture has been widely reported in past literature (Fortier et al. 1988; Herath et al. 2006), although these reports often lack crucial details allowing for their methods to be replicated. Here we detail a protocol for the isolation, culture and characterisation of pure, distinct populations of endometrial epithelial and stromal fibroblast cells, aiming to provide a useful model for the investigation of endometrial cell-mediated inflammation.

## Methods

### Cell isolation and culture

Bovine female reproductive tracts were collected at a local abattoir within 15 min of slaughter. Collected tracts were identified as being free from disease and visible infection. Only tracts in the early luteal stage of oestrous (as identified by the presence of a stage 1 corpus luteum on one ovary) were used. Details of age and breed were also recorded at the time of collection.

The external surface of the tract was washed in 70% industrial methylated spirits (IMS) and the uterine horn ipsilateral to the corpus luteum was opened longitudinally with sterile scissors. The exposed endometrium was washed in PBS (Gibco, Paisley, UK) supplemented with 50 IU/ml penicillin, 50 IU/ml streptomycin (Gibco) and 2.5 g/ml amphotericin B (Sigma-Aldrich, Poole, UK). The endometrial surface was dissected using a sterile scissors and forceps. Dissection was performed at the abattoir with the tissue harvested into a 50 ml tube containing 20 ml of RPMI (Gibco) supplemented with antibiotics as above. Samples were transported to the laboratory at room temperature within 90 min of collection.

On arrival at the laboratory, tissue was washed twice in room temperature HBSS (Gibco, Madison, WI, UK) supplemented with antibiotics. The tissue was then chopped into fine pieces (approximately 1-3 mm^3^ in size) using a sharp scissors and scalpels. The tissue was then incubated in HBSS with antibiotics at 37 °C for 10 mins in order to slowly return the tissue temperature to 37 °C. HBSS was removed before the addition of 20 ml digestive solution (pre-warmed to 37 °C). Digestive solution (100 ml) consisted of 375 BAEE units of trypsin-EDTA (Sigma-Aldrich), 50 mg collagenase II (Sigma-Aldrich), 100 mg bovine serum albumin (BSA)(Sigma-Aldrich) and 10 mg DNase I (Sigma-Aldrich) made up to 100 ml with HBSS and 0.2 μm sterile filtered (Sartorius, Göttingen, Germany). Samples incubated with digestive solution were placed in a 37 °C shaking incubator at 150 rpm for 1 h.

The resulting mixture was filtered through a 70 μm nylon mesh cell strainer (Becton, Dickinson, Flintshire, UK) to remove any cell debris with the filtrate being collected in a tube containing HBSS with 10% FBS (Gibco). This solution was then passed through a 40 μm cell strainer (Pluristrainer, Pluiselect, Leipzig, Germany) to isolate the stromal cells. As the epithelial cells were too large to pass through the 40 μm cell strainer they remained on the strainer. The epithelial cells could then be isolated by turning the 40 μm cell strainer upside down and washing the epithelial cells into a clean 50 ml tube using 10 ml of HBSS supplemented with 10% FBS. Cells were then pelleted by centrifugation at 700 x g for 10 min. Red blood cells were lysed by re-suspending the pellet in 1 ml sterile water (Gibco) before addition of 4 ml of complete growth media. This consisted of RPMI 1640 supplemented with antibiotics as above, 10% FBS and 1X insulin, transferring, selenium supplement with ethanolamine (ITS-X) (Gibco).

Cells were pelleted as before and re-suspended in complete growth media. Cells were counted using a haemocytometer with trypan blue (Sigma-Aldrich, Poole, UK) staining to distinguish live from dead cells. Cell counts were adjusted to 3 × 10^5^ cells/ml and 8 × 10^5^ cells/ml for epithelial and stromal fibroblasts respectively and 1 ml was were seeded into 75cm^2^ vented tissue culture flasks (Greiner Bio-One, Kremsmünster, Austria) with complete growth medium, which was stored in a 37 °C incubator in 5% CO_2_. Cells were allowed to reach 85% confluency before plating, with media changing every 48 h. Visual examination of cell morphology under the light microscope was used to check purity of cultures in addition to characterisation based on expression of differing cytoskeletal proteins.

### Isolation and culture of PBMCs from whole blood

Bovine peripheral blood mononuclear cells (PBMCs) were isolated from whole blood samples collected in 9 ml vacutainers containing Heparin anticoagulant (Greiner Bio-One, Kremsmünster, Austria). Whole blood was diluted 1:1 with HBSS and mixed gently before 25 ml was layered onto 15 ml of sterile Ficoll-paque density gradient medium (Fisher Scientific, New Hampshire, US) in a 50 ml tube. The samples were then centrifuged at 800×g for 25 mins with the centrifuge break turned off. The mononuclear cells were carefully removed with a pastette into a new 50 ml tube and made up to 10 ml with wash buffer (HBSS and 5% FBS). Cells were pelleted by centrifugation at 300×g for 5 mins. Any red blood cells carried over during the separation process were lysed by re-suspending the cells in red blood cell lysis buffer (Sigma-Aldrich). The cell pellet was then re-suspended in complete RPMI media (containing 10% FBS and 1% Pen-Strep) and cells were counted using a haemocytometer and trypan blue staining. Cell were then seeded at appropriate densities in tissue culture plates containing media. The cells were incubated at 37 °C with 5% CO_2_.

### Processing of endometrial samples for histological analysis

Endometrial biopsies were removed from 10% neutral buffered formalin (Sigma-Aldrich) and immersed in 70% IMS for 24 h before overnight paraffin wax embedding in Surgipath cassettes (Leica Biosystems, Newcastle, UK). Embedded biopsies were sectioned at 3 μm, dewaxed by immersion in histoclear (National Diagnostics, Atlanta, GA, USA) and rehydrated by immersion in 100%, 90%, 80% and 75% ethanol (Sigma-Aldrich). Haemotoxylin (Leica Biosystems) was applied to the tissue sections for 10 min before immersion in Scott’s tap water (30 g magnesium sulphate (Sigma-Aldrich) and 2 g sodium bicarbonate (Sigma-Aldrich) in 3 L tap water) for 5 mins. Tissue sections were progressively dehydrated by a reversal of the ethanol immersion steps used previously and mounted with Surgipath mounting medium (Leica Biosystems). Slides were examined using an Olympus BX51 upright microscope (Leica Biosystems).

### Immunofluorescent staining of endometrial cell cytoskeletal proteins

Epithelial or stromal cells were seeded at a density of 1 × 10^5^ cells/ml into 24-well tissue culture plates in complete growth medium, allowing one well each for cytokeratin staining (expressed by epithelial cells), vimentin staining (expressed by stromal cells), an IgG isotype control and an unstained well, and allowed to rest for 24 h. Growth medium was removed, the cell monolayer was rinsed three times with PBS and fixed with 500 μl 10% neutral-buffered formalin at room temperature for one hour. Formalin was removed and the cells washed three times with PBS before the primary antibody was added. Mouse anti-human cytokeratin antibody (Dako/Agilent Technologies, Santa Clara, CA, USA), mouse anti-human vimentin antibody (Sigma-Aldrich) or IgG1 isotype control (Sigma-Aldrich) were applied as appropriate using 400 μl per well of a 1:100 dilution in PBS. Cells were incubated at 4 °C with rocking overnight. An FITC-conjugated polyclonal goat anti-mouse secondary antibody (Abcam, Cambridge, UK)) was applied to all wells in a 1:250 dilution, incubating at 37 °C for 1 h. After rinsing three times in PBS, DAPI stain (Sigma-Aldrich) to detect nuclei was applied in a 1:250 dilution for 10 min at 37 °C. An Olympus IX81 inverted microscope was used to visualise immunofluorescent stained cells, with positive cytokeratin staining in the absence of vimentin staining taken to indicate a pure endometrial epithelial cell culture and the inverse being characteristic of stromal cells.

### Immunocytochemistry

Epithelial or stromal fibroblast cells were seeded at a density of 1 × 10^5^ cells/ml or 0.5 × 10^5^ cells/ml respectively into wells of the Millicell EZ SLIDE 8 well glass culture slides (Millipore), and allowed to rest for 24 h. Cells were washed three times in PBS before being fixed in ice-cold methanol for 10 mins at room temperature. Cells were then washed a further three times in PBS before incubation in 3% H_2_O_2_ (Sigma-Aldrich)) for 20 mins at room temperature, followed by blocking in 10% BSA (VWR) for 1 h at room temperature. Cells were then incubated overnight with mouse anti-human cytokeratin antibody, mouse anti-human vimentin antibody or IgG1 isotype control using 100 μl per well of a 1:100 dilution in PBS. Cells were then washed three times in PBS before incubation with the secondary antibody (Dako) for 30 mins at room temperature. Cells were washed a further three times before incubation with the chromogen diaminobenzidin (DAB) (Dako) for 10 mins. Cells were washed a further three times before being mounted with Surgipath mounting medium. Slides were examined using an Olympus BX51 upright microscope.

### Western blotting

Epithelial or stromal fibroblasts cells (1 × 10^6^ cells/ml) were harvested in RIPA buffer supplemented with the protease inhibitors leupeptin (2 μg/ml), aprotonin (2 μg/ml), sodium orthovanadate (4 μg/ml) and phenylmethylsulphonyl fluoride (100 μg/ml). Protein quantification was performed using BCA Protein Assay Reagents (Pierce Biotech) according to the manufacturer’s instructions. A standard curve was generated each time with BSA standards and the protein concentration of each sample was calculated from this. From these values, protein samples were normalised relative to the sample of lowest concentration in 20 μl dilutions. Loading buffer (4X Tris-glycine SDS β-mercapthenol) was added to each sample (5 μl) and boiled at 95 °C for 5 min.

Prior to SDS-PAGE a 15% running gel, topped with a 4% stacking gel, were prepared. The gels were loaded into the running tank and 1X Running buffer (0.025 M Tris, 0.192 M glycine, 0.1% SDS) was added and poured into the upper and lower reservoirs of the apparatus. The samples and 3 μl molecular marker was used per gel. The gel was run at 110 V for 45 mins. After the run, the stacking gel was removed and the running gel retained for transfer to PVDF membranes.

Transfer buffer (0.048 M Tris, 0.039 M glycine, 20% methanol, 0.00375% SDS), PVDF membrane (0.2 μm pore size) and blotting pads were prepared. The transfer membrane were cut to correct size and activated by immersion in methanol for 30 s and then in dH_2_0 for 2 mins. The membrane and blotting pads were then soaked in transfer membrane for 5 mins. Once the gel had ran, it was removed from the gel apparatus and placed in transfer buffer for 5 mins. The gel was then transferred to the transfer machine where it was sandwiched between blotting pads and a transfer membrane. The transfer machine was connected to a voltmeter and run at 10 V (250 mA) for 20 mins.

Once the membrane was removed from the transfer machine, it was blocked to prevent non-specific binding of antibody with 5% milk in PBS tween (PBS-T) for 1 h. After blocking, the primary antibody was added and the blot was left rocking in the cold room overnight. Primary antibody was diluted to a concentration of 1:1500 in 30mls of PBS-T. Before adding the secondary antibody, the membrane was washed for 5 mins three times in PBS-T. Secondary antibodies were applied (1:5000 dilution in 5% milk) and the blot was left on the rocker for 1 h at room temperature. The blot was then washed again for 5 mins three times, before being incubated in ECL (Biorad) and visualised on the Biorad ChemiDoc MP System.

### Cell treatments

For stimulations of epithelial and stromal fibroblast populations, cells were plated at a density of 1.5 × 10^5^ cells/ml in a 24 well plate and left to rest for 24 h before stimulation. Following the 24 h rest period, medium was removed and replaced with control medium or medium containing 2 μg/ml LPS (Enzo Life Sciences, Exeter, UK) for 3, 6, 12 or 24 h. Following stimulation cells were lysed in TRIzol reagent (Thermo Fisher Scientific, Paisley, UK) for mRNA extraction and stored at −80 °C.

### Quantitative PCR (qPCR)

Total RNA was extracted using TRIzol reagent according to manufacturer’s instructions. RNA quantity was calculated using ND-1000 NanoDrop spectrophotometer (Thermo Fisher Scientific). One microgram of RNA was reverse transcribed into cDNA using the OmniScript kit (Qiagen, Crawley, UK) with Oligo (dT) primers (Qiagen) according to the manufacturer’s instructions. cDNA solutions were diluted 1:10 before qPCR analysis.

qPCR was performed using the following primers: *IL-6F* CAGGAACGAAAGAGAGCTCCA; *IL-6R* AATGGAGTGAAGGCGCTTGT; *IL-8F* ATTCCACACCTTTCCACCCC; *IL-8R* TTGCTTCTCAGCTCTCTTCACA; *S100A8F* ATTTTGGGGAGACCTGGTGG; S100A8R GCTTCCAGGCCCACCTTTAT; *S100A9F* GCTTCTCGGCTTGGTAGGAG; S100A9R CCTCCATTTTCCCGCCTTCT; *H3F3AF* CATGGCTCGTACAAAGCAGA; *H3F3AR* ACCAGGCCTGTAACGATGAG. Primers were designed using the Primer BLAST software to be intron spanning where possible. Optimal primer concentrations (*IL8*: 300 nM, *IL6*: 500 nM, *S100A8*: 300 nM, *S100A9*: 500 nM *H3F3A*: 100 nM) were assessed by titrating different final concentrations and dissociation curves were examined for the presence of a single product.

Quantitative PCR was run using the PowerUp SYBR Green Master Mix (Thermo Fisher Scientific) on a StepOnePlus Real-Time PCR System (Thermo Fisher Scientific) using the following parameters: 95 °C for 20 s, followed by 40 cycles of 95 °C for 3 s and 60 °C for 30 s and a final amplicon dissociation step at 95 °C for 15 s, 60 °C for 1 min and 95 °C for 15 s. *H3F3A* was found to be the most stably expressed reference gene from a panel of reference genes tested using GeNorm software and was subsequently used to generate normalized relative expression values (Vandesompele et al. 2002).

The HotStar Master Mix PCR kit (Qiagen) was used to carry out a PCR reaction to detect transcription of the protein tyrosine phosphatase, receptor type C (PTPRC) gene, encoding the pan-leukocyte marker CD45, within endometrial cell cultures. A 10 μl reaction volume contained 0.3 μl endometrial cell cDNA, 1X CoralLoad reaction buffer, 200 μM dNTP solution, 0.3 μl HotStarTaq polymerase enzyme and 300 nM PTPRC-specific primers (Forward: TGCAACCGCTCTCTCAACCATA, Reverse: CTTGCTTGGCTTTGCTGGATCT), with nuclease free water making up the remainder. cDNA prepared from bovine PBMCs was used as a positive control for *PTPRC* amplification. The constitutively expressed ribosomal protein S9 *(RPS9F* GCGTCTGTTCGAAGGTAATGC; *RPS9R* AAGTCGATGTGCTTCTGCGA) was amplified from all samples to ensure poor cDNA quality did not account for a lack of amplification. A non-template control without cDNA was run for both gene assays. The PCR reaction was carried out in a Techne Prime thermocycler (Bibby Scientific, Staffordshire, UK) using the following thermocycling conditions: 95 °C for 5 min and 40 cycles of 95 °C for 30 s, 60 °C for 1 min, 72 °C for 1 min. Results were assessed by presence or absence of a DNA product of expected size on a 2% agarose gel after electrophoresis.

### Data analysis

For gene expression analysis, qPCR data was converted to gene expression fold changes using the 2^-ΔΔCq^ method (where Cq represents the quantification cycle) (Schmittgen and Livak 2008). H3F3A was used as a reference gene following GeNorm analysis. Statistical analysis of qPCR data was performed using a non-parametric Kruskall-Wallis test with Dunns multiple comparison post-hoc test as implemented in Graphpad Prism 7 software.

## Results

### Optimisation of tissue isolation

Dissection was performed on the uterine horn ipsilateral to the corpus luteum with the uterine horn dissected from the bifurcation of the uterine horns to the top of the uterine horn (Supplementary Figure 1A). The oestrous cycle stage of each tract was determined by examining the ovaries and identifying the presence of a stage I corpus luteum (Supplementary Figure 1B). Tracts in the early luteal phase of oestrous were chosen for basal levels of progesterone which would therefore not impact on inflammatory mediator production (Butts et al. 2007; Stites and Siiteri 1983). Tracts were collected from healthy cows who were on average 91.6 months old (7.6 years) and predominantly Holstein-Friesians (Supplementary Figure 1 C-D).

Dissection of the upper functional layer of the endometrium was optimised using a curved dissection scissors and forceps. The endometrial lining was dissected in thin strips using a curved scissors and forceps (Supplementary Figure 1G). The forceps was used to hold the edge of the endometrial lining while the curved scissors passed underneath the endometrial lining, cutting away the fibres that tie it to the lower functional layer composed of mainly stromal fibroblasts. Harvested tissue was immediately stored in transport media. We also investigated the use of a curette, an instrument with a sharp loop at the end of a long handle commonly used in human surgery to sample the endometrium. Tissue isolation using a curette involves running the loop over the endometrium to harvest the epithelial layer. While dissection with the curette was faster and required less skill, we found we recovered higher cell yields (particularly epithelial cells) by dissecting with the scissors, possibly due to less tissue recovered using the curette (Supplementary Figure 1H).

Histology images taken before and after dissection (Supplementary Figure 1 E, F) show that the uppermost functional layer containing the epithelium is removed during dissection. In the intact endometrium (Supplementary Figure 1E) the luminal epithelium forming a barrier overlying the stroma is observed. The stroma also contains glandular epithelium and spiral arterioles which maintain the blood supply.

### Optimisation of tissue transport

Our initial protocol for cell isolation and transport involved tract collection at the abattoir and transportation on ice to the laboratory (transport time taking approximately 1–1.5 h). However cell viability using this approach was low. To improve cell viability, we harvested the endometrial tissue at the abattoir. Collected tracts were cleaned with 70% IMS and the exposed endometrium washed with PBS supplemented with 50 IU penicillin, 50 IU streptomycin and 2.5 μg/ml amphotericin B prior to dissection and transported the dissected tissue back in culture media (RPMI supplemented with 10% FBS). This resulted in problems with bacterial contamination of our isolated cultures, probably due to the lack of a proper aseptic environment while performing the dissection in the abattoir. To address this issue we investigated several different transport conditions. Tissue was transported in media with or without FBS and with or without antibiotics. The tissue was also transported on ice or at room temperature. Use of media lacking FBS but containing antibiotics and transported at room temperature was found to result in high yields of viable cells with no contamination.

### Cell isolation by differential size filtration

Disruption of the harvested tissue was performed using both mechanical dissociation and by enzymatic dissociation. Mechanical dissociation was performed by using a sharp scissors to chop the tissue finely (1-3 mm in size) before proceeding to enzymatic dissociation. An enzymatic digestion solution had previously been optimised containing trypsin (375 BAEE units), collagenase (50 mg), BSA (100 mg) and DNase (10 mg) made up to 100 ml HBSS (Fortier et al. 1988; Herath et al. 2006). Low concentrations of trypsin (375 BAEE units) were used to avoid damage to the cells. Trypsin concentration was determined using activity units rather than volume due to large batch-batch variation in trypsin activity (MacKintosh et al. 2013). We found collagenase was able to digest the extracellular matrix, separating the epithelial and stromal cells but did not dissociate epithelial cells, allowing them to remain in viable clusters or islands. DNase was used to remove DNA released from lysed cells which can promote re-aggregation of dissociated cells. Incubation times ranging from 1 to 2 h have been reported (Cheng et al. 2003; Fortier et al. 1988; Herath et al. 2006). We found incubation at 37 °C for 1 h in a shaking incubator was sufficient for cell dissociation.

The initial protocol we used for isolation of pure populations of epithelial and stromal fibroblast cells involved adding the total cell isolate to a tissue culture flask and allowing 24 h for stromal fibroblast cells to adhere before the epithelial cells still in suspension were harvested and re-plated in a new flask (Herath et al. 2006). However, this approach did not yield pure populations of either cell type and so we separated by size using filters to address this problem. Stromal cells easily passed through 40 μm filters while epithelial islands were retained. Flipping the filters and backwashing the epithelial cells allows for the easy isolation of pure epithelial populations. Any residual stromal cell contamination of these epithelial populations could be removed in the flasks by accutase, as stromal cells lift in the first 5 min of accutase treatment while epithelial cells take a minimum of 15 min to lift. Removal of red blood cells was performed by the addition of 1 ml sterile water to the cell pellet followed by gentle pipetting. Initial attempts at removing red blood cells included the use of red blood cell lysis buffer and vortexing for 30 s. However, this resulted in a higher proportion of epithelial or stromal cell death.

### Cell culture, characterisation and cryopreservation

**Fig. 1:**
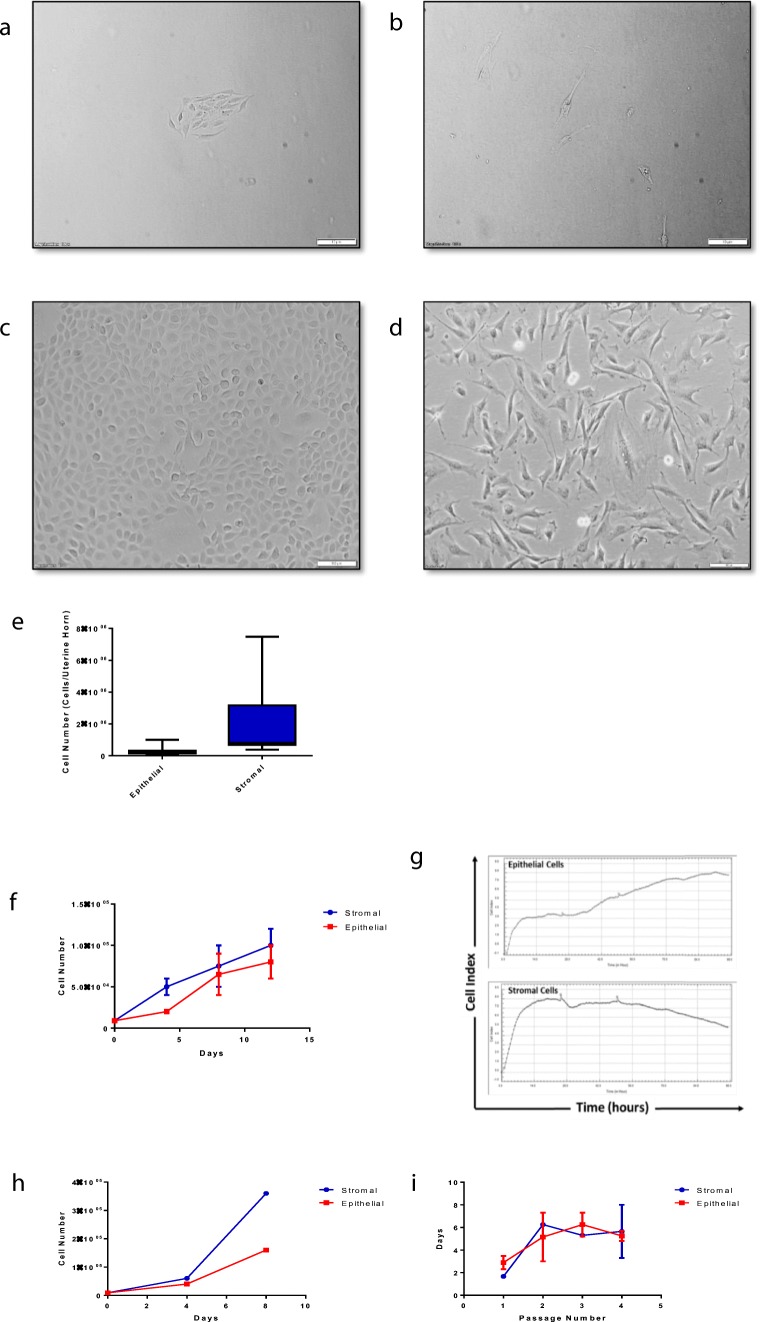
Endometrial epithelial and stromal fibroblast cells exhibit distinct morphological features. Representative images of endometrial epithelial and stromal cells showing distinct morphological differences between the two cell populations. **a** Small cluster of epithelial cells visible after 2 days of culture. **b** Stromal cells appear scattered throughout the flask following 2 days of culture. **c** Pure population of endometrial cells following 14 days of culture. **d** Pure population of endometrial stromal fibroblast cells following 6 days of culture. Scale bar indicates 100 μm. Images were taken using an Olympus IX81 inverted microscope with camera attached. **e** Average epithelial cell yield isolated from tissue was 3.08 × 10^5^ ± 0.66 × 10^5^, average stromal cell yield was 1.88 × 10^6^ ± 5.44 × 10^5^ (*n = 15).* Following isolation cell pellets were counted under a light microscope using a haemocytometer and trypan blue staining. Values presented are mean cell counts per uterine horn ± SEM. **f** Growth curve showing the increase in cell populations over 12 days of culture. Endometrial epithelial (▪) and stromal (•) cells were plated at a density of 9 × 10^3^ cells into six-well tissue culture plates and counted over the course of 12 days using a haemocytometer and trypan blue staining. Values presented are mean cell count ± SEM. **g** Growth curve showing the increase in cell populations over a period of 96 h as measured by the xCelligence real time cell analyser. **h** Growth curve showing increase in cell populations following cryopreservation. **i** Doubling time across consecutive passages of both endometrial epithelial (▪) and stromal cells (•). Cells were plated at a density of 9 × 10^3^ cells into six-well tissue culture plates and over 16 days of culture, cells were detached using trypsin, diluted in trypan blue and counted using a haemocytometer before being reseeded into the 6 well plate at the same density

During initial isolations, flasks were coated with 0.1% gelatin to promote cell adherence. We also examined the tissue culture growth media and its components. Using different amounts of FBS and trying supplements such as Insulin-Transferrin-Selenium (ITS-X) we found that the optimal growth media contained 10% FBS and 5% ITS-X. Purified cell populations were identified by morphology. Epithelial cells grow in small islands that ultimately combine to form a confluent monolayer (Fig. 1a, c) while stromal fibroblast cells were found scattered throughout the flask (Fig. 1b, d). Using the above process, stromal fibroblast cells (1.88 × 10^6^ cells per uterine horn) and epithelial cells (3.05 × 10^5^ cells per uterine horn) of >95% viability were isolated following this isolation process (Fig. 1e). The ability of both cell populations to proliferate in culture is shown by the cell culture images and growth curve in Fig. 1, with both cell populations maintaining a steady increase in cell numbers over the first 12 days of culture (Fig. 1f-g).

We next investigated whether endometrial cells could be cryopreserved or passaged using standard protocols. The growth curve in Fig. 1(h-i) shows that both cell populations recover well from cryopreservation and continue to proliferate in culture. No morphological differences were observed in the cryopreserved cells after reconstitution. Beyond two passages morphological differences begin to appear in both cell populations and both populations become slower to proliferate, taking approximately 5–6 days to double their population as opposed to 2 days after just one passage.

**Fig. 2:**
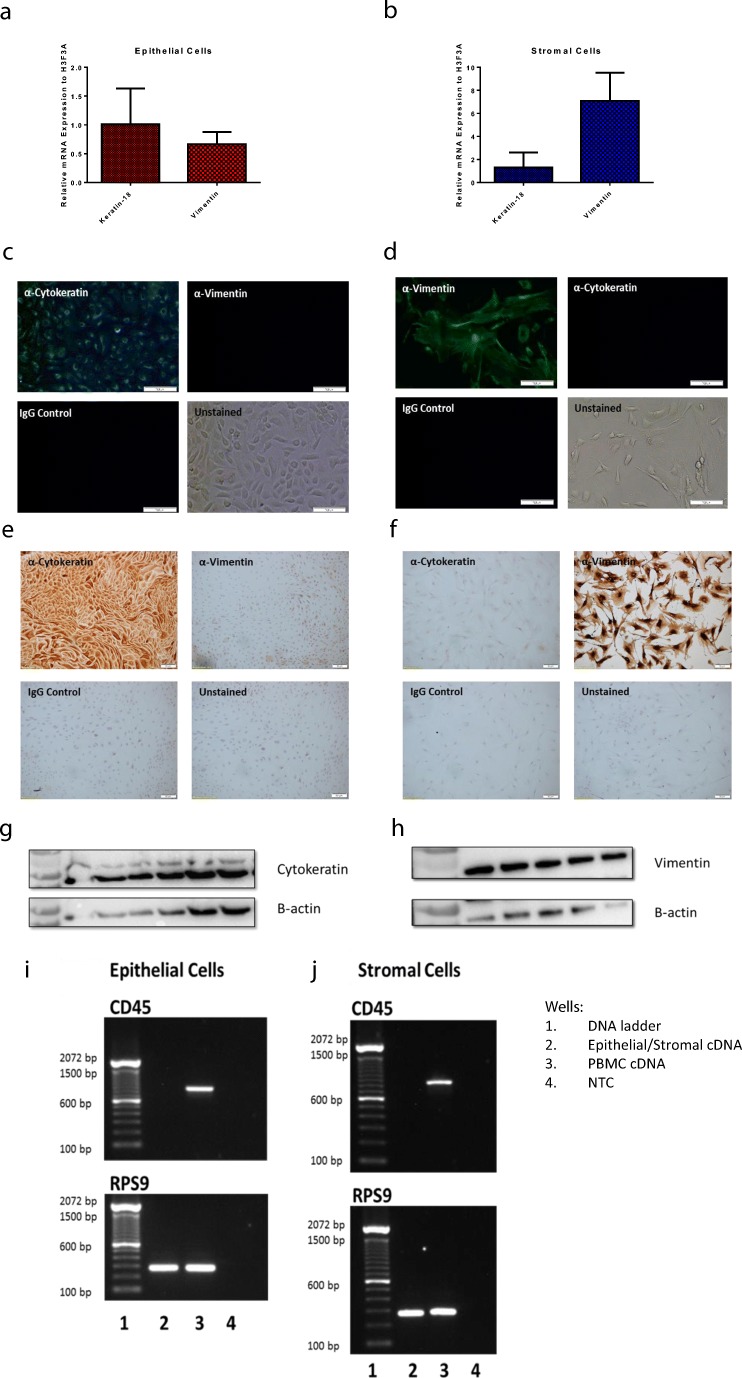
Endometrial cell populations can be identified based on their differential expression of cytoskeletal proteins. Relative keratin-18 and vimentin mRNA gene expression in (**a**) endometrial epithelial and (**b**) stromal cell populations. Immunofluorescence staining of (**c**, **e**) endometrial epithelial and (**d**, **f**) stromal fibroblast cells with antibodies against cytoskeletal proteins cytokeratin and vimentin. Scale bar indicates 100 μm. Western blotting staining of (**g**) endometrial epithelial and (**h**) stromal fibroblast cell lysates with antibodies against cytokeratin and vimentin. Gel electrophoresis image of PCR products for the *PTPRC* gene (which encodes CD45 protein) and *RPS9* (reference gene) from (**i**) endometrial epithelial and (**j**) stromal fibroblast cells. cDNA from PBMCs was included as a positive control

Purity of the epithelial and stromal cells populations was demonstrated by the expression of cell specific cytoskeletal proteins (Fig. 2). Epithelial cells express cytokeratin while stromal cells express vimentin (Franquemont et al. 1991; Wonodirekso et al. 1993) (Fig. 2(a & b). Immunofluorescent, immunocytochemical and western blotting staining for the cytoskeletal proteins cytokeratin and vimentin confirmed qPCR data (Fig. 2c-h).

As we aim to use our primary culture model to investigate the innate immune responses of endometrial cells we needed to be certain that there was no immune cell contamination of our cultures. A PCR assay for *PTPRC*, the gene that encodes CD45, a marker of immune cells was optimised. PBMC cDNA was used as a positive control. The housekeeping gene *RPS9* was included to ensure cDNA quality. Cultures were negative for CD45 (Fig. 2i-j) indicating absence of immune cell contamination, again confirming the purity of our endometrial cell populations.

### Expression of inflammatory mediators

**Fig. 3:**
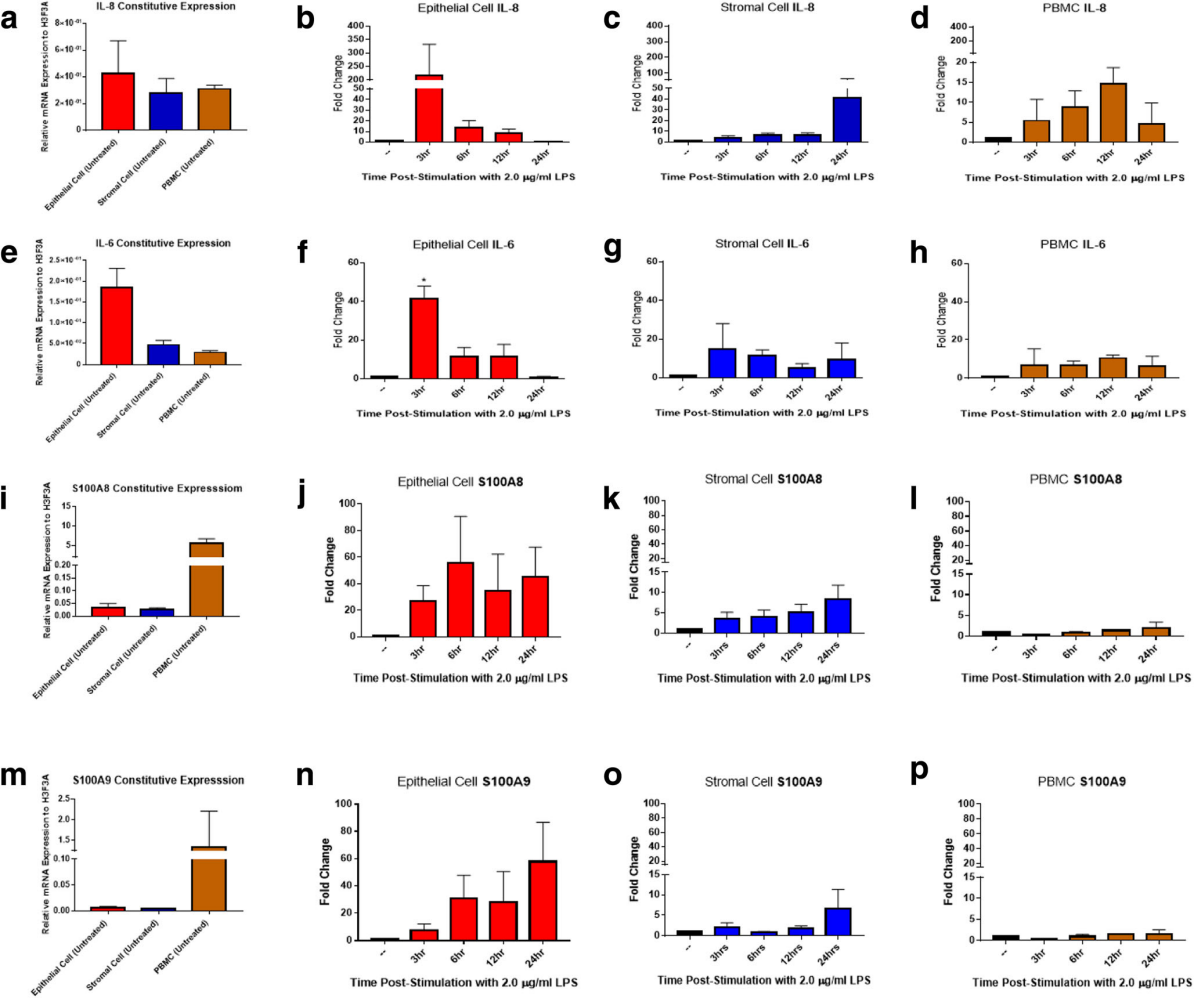
Endometrial epithelial and stromal fibroblast cells express *IL6*, *IL8, S100A8* and *S100A9* mRNA in response to LPS stimulation. Following an LPS time-course stimulation, endometrial cells were harvested in TRIzol reagent, mRNA was extracted and reverse transcribed to cDNA before *IL8, IL6, S100A8* and *S100A9* gene expression was examined by qPCR. (**a**-**d**) *IL8* mRNA levels in endometrial cells and PBMCs following stimulation (*n = 3*). (**e**-**h**) *IL6* mRNA levels in endometrial cells and PBMCs following stimulation (*n = 3*). (**i**-**l**) *S100A8* mRNA levels in endometrial cells and PBMCs following stimulation (*n = 3*). (**m**-**p**) *S100A9* mRNA levels in endometrial cells and PBMCs following stimulation (*n = 3*)

Having generated pure endometrial cell populations, a time course of the response of these cells to the microbial PAMP, LPS, was performed to investigate their ability to detect and respond to inflammatory stimuli compared to the more classically defined immune cells present in PBMCs. Results indicate that epithelial and stromal fibroblast populations display significantly divergent response profiles. Constitutive expression of *IL8* and *IL6* mRNA, as quantified using qPCR, was higher in epithelial cells than stromal fibroblasts or unmatched PBMCs (in which levels were comparable) (Fig. 3a and e). *IL-8* mRNA was significantly induced at earlier time-points (3 h post-stimulation) in epithelial cells while stromal cells show a higher induction of *IL8* at 24 h post-stimulation, similar to PBMCs which demonstrate peak *IL8* mRNA production at 12 h post stimulation (Fig. 3b-d). Induction of *IL6* mRNA demonstrated a similar pattern, with peak production of *IL6* occurring in epithelial cells 3 h post stimulation, and lower induction of *IL6* by stromal fibroblast cells and PBMCs at the same time-points (Fig. 3f-h). In contrast, constitutive expression of the antimicrobial genes *S100A8* and *S100A9* was highest in PBMCs compared to the low levels detected in endometrial cells (Fig. 3i and m). However, upon stimulation with LPS endometrial cells were more responsive compared to PBMCs, with epithelial cells demonstrating stronger induction of both *S100A8* (Fig. 3j-l) and *S100A9* (Fig. 3n-p) compared to stromal fibroblasts.

## Discussion

Infection of the endometrium by microbial and viral pathogens can result in pathological inflammation that is harmful to cow health and agricultural sustainability (Bromfield et al. 2015; Sheldon et al. 2008). Epithelial and stromal fibroblast cell populations play important but distinct roles in innate defence of the endometrium. Understanding the inflammatory pathways and mechanisms of response in endometrial cells is crucial to developing therapeutics targeted towards the specific inflammatory insult, thereby improving cow health and fertility and ultimately increasing profitability. Elucidating these inflammatory pathways requires a reliable and well characterised model in order to elucidate which cell type is contributing to the inflammation.

Bovine endometrial epithelial cells and stromal fibroblasts have been grown with varying degrees of success since the first reports describing their isolation (Fortier et al. 1988; Herath et al. 2006). Our optimised protocol gives consistently pure and viable populations of epithelial and stromal cells, confirmed by the positive staining for their respective cytoskeletal proteins cytokeratin and vimentin and by the easily observed differences in cell morphology. We have also confirmed the absence of immune cell contamination by PCR for *PTPRC* which encodes CD45, the immune cell surface marker.

Here we describe several key modifications to previously published protocols. Dissection and harvesting of the endometrium occurs immediately after recovering the tract in the abattoir as opposed to transporting the tract back to the laboratory on ice. We found this greatly improved cell viability and numbers of cells recovered. Separating cells based on size using differential filtering also greatly improved the purity of the distinct cell populations. We found the brand of cell strainer used impacted on cell recovery. In addition, any residual stromal cell contamination of our epithelial cultures was removed with accutase treatment based on differential lifting time. Finally, supplementing the growth media with insulin, transferrin, selenium solution (ITS-X) also had a marked impact on the growth of our cell populations in culture.

The induction of inflammatory cytokine mRNA in response to LPS stimulation confirms that isolated and cultured endometrial cells retain the ability to respond to bacterial stimuli in-vitro. While the induction of the cytokines *IL8* and *IL6* by endometrial cells has been previously reported (Cronin et al. 2012; Healy et al. 2015), their induction here, in addition to the antimicrobial *S100A8* and *S100A9*, confirms that our improved protocol for isolating and culturing endometrial cells provides a practical option for the study of bovine inflammatory responses in-vitro.

This model will facilitate investigations into the production of immune molecules such as cytokines, chemokines, HDPs and acute phase proteins under defined inflammatory conditions. It is our aim that the protocol detailed here will provide a useful basis for others wishing to establish a similar model within their laboratory.

## Electronic supplementary material

Supplementary Fig. 1 **Optimisation of dissection technique.** Collection of female reproductive tracts and isolation of endometrial tissue was initially optimised using a curved scissors dissection or dissection with a curette. **(A).** Image of a uterus following dissection of the endometrial lining of the uterine horn ipsilateral to the corpus luteum. **(B)** Example of ovary staging for tract collection. A stage 1 corpus luteum (or corpus hemorrhagicum) is clearly visible. **(C)** Histogram detailing the age categories of cows from which endometrial samples were obtained. **(D)** Details of the breed of cows from which endometrial samples were obtained. **(E & F)** Representative histological image of the endometrium before and after dissection of the epithelial layer respectively. Lum=endometrial lumen, LE=luminal epithelium, Str=stroma, GE=glandular epithelium. Scale bar indicates 200μm. **(G)** Image of tissue isolated from the functional layer of the endometrium. **(H)** Comparison of tissue weight isolated using either a scissors or curette for dissection (PDF 499 kb)

## Data Availability

The datasets analysed during the current study are available from the corresponding author on reasonable request.
