# Supplementary figures and images for: Improved filtration method to isolate pure populations of primary bovine endometrial epithelial and stromal cells for immunological studies
Source: Vet Res Commun. 2020 Feb 21;44(1):29–39. doi: 10.1007/s11259-020-09770-3 (PMC7039867; doi:10.1007/s11259-020-09770-3)

Additional File 1. Optimisation of dissection technique.

A.

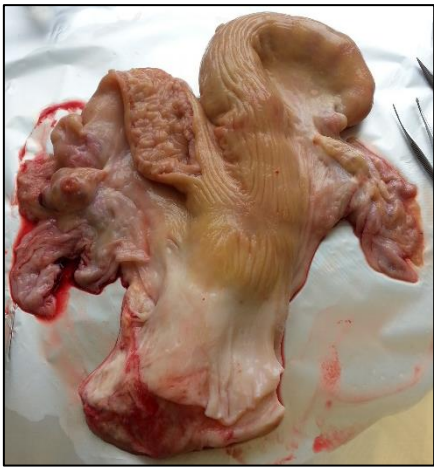

B.

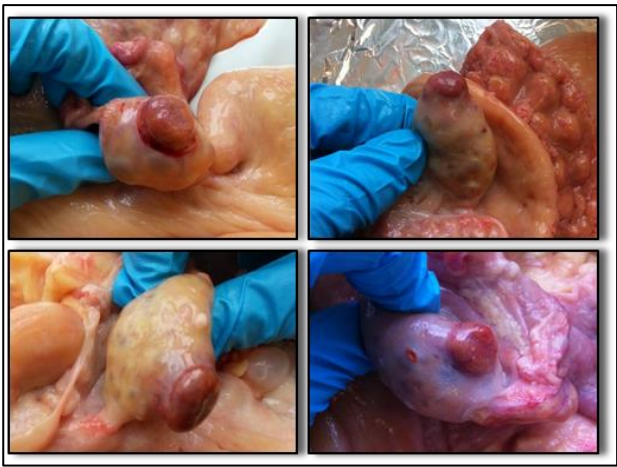

C.

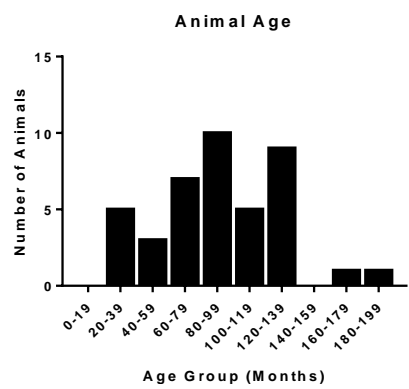

D.

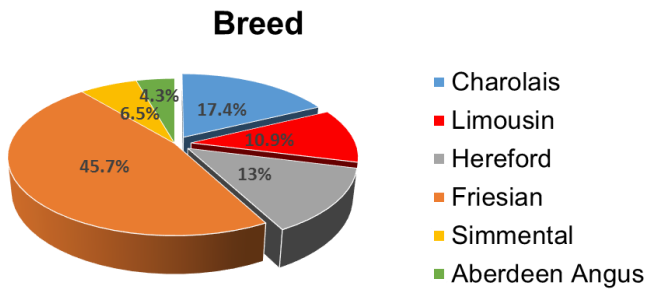

E.

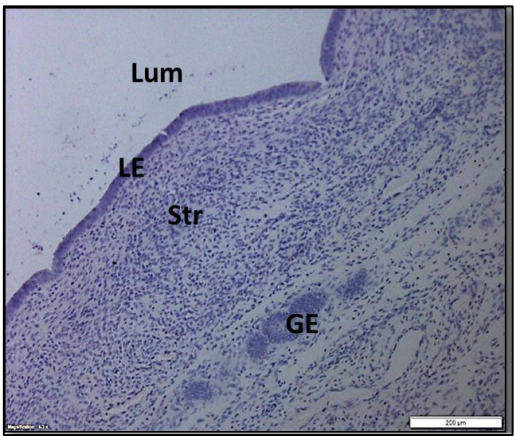

F.

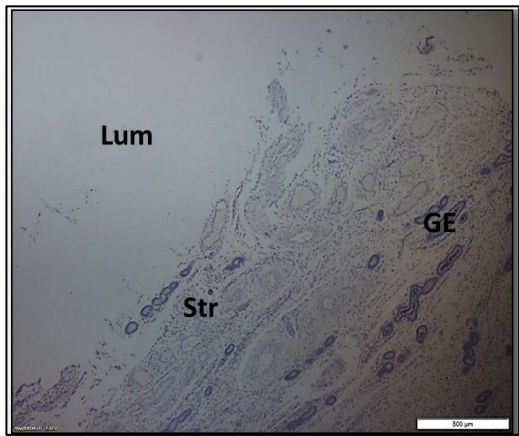

G.

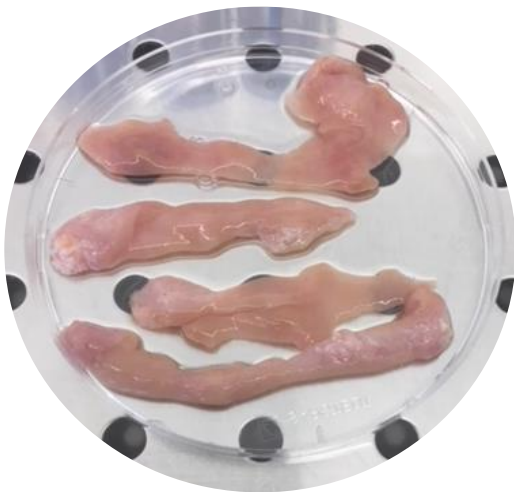

H.

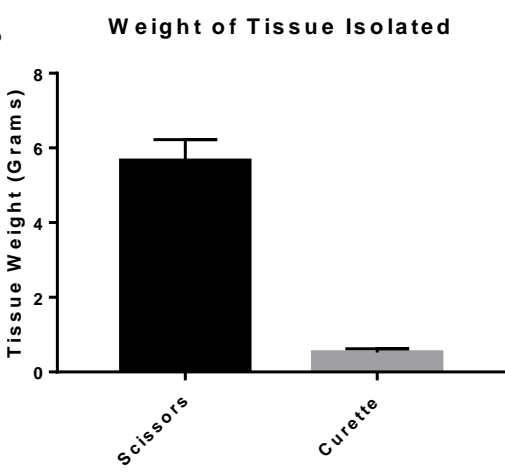

Supplement: Supplementary file 1 — Optimisation of dissection technique. Collection of female reproductive tracts and isolation of endometrial tissue was initially optimised using a curved scissors dissection or dissection with a curette. (A). Image of a uterus following dissection of the endometrial lining of the uterine horn ipsilateral to the corpus luteum. (B) Example of ovary staging for tract collection. A stage 1 corpus luteum (or corpus hemorrhagicum) is clearly visible. (C) Histogram detailing the age categories of cows from which endometrial samples were obtained. (D) Details of the breed of cows from which endometrial samples were obtained. (E & F) Representative histological image of the endometrium before and after dissection of the epithelial layer respectively. Lum=endometrial lumen, LE=luminal epithelium, Str=stroma, GE=glandular epithelium. Scale bar indicates 200μm. (G) Image of tissue isolated from the functional layer of the endometrium. (H) Comparison of tissue weight isolated using either a scissors or curette for dissection (PDF 499 kb) [file 11259_2020_9770_MOESM1_ESM.pdf]
